# Effect of trauma life support training on patient outcomes: a systematic review and meta-analysis

**DOI:** 10.1186/s13049-026-01549-w

**Published:** 2026-01-20

**Authors:** Zaynab Nakhid, Martin Gerdin Wärnberg, Johanna Berg, Kapil Dev Soni, Monty Khajanchi, Deepa Kizhakke Veetil, Siddarth Daniels David

**Affiliations:** 1 Department of Global Public Health, Karolinska Institutet, Stockholm, Sweden; 2 Perioperative Medicine and Intensive Care, Karolinska University Hospital, Solna, Sweden; 3 Department of Emergency Medicine, Skåne University Hospital, Malmö, Sweden; 4 JPN Apex Trauma Center, All India Institute of Medical Sciences, New Delhi, India; 5 GSMC & KEM Hospital, Mumbai, India; 6 World Health Organization Collaborating Centre for Emergency, Critical and Operative Care, Program for Global Surgery and Trauma, The George Institute for Global Health, Delhi, India; 7 Max Super Speciality Hospital, Dwarka New Delhi, India

**Keywords:** Trauma, Trauma care, Training, ATLS, PTC, Mortality, Morbidity, Return to work

## Abstract

**Background:**

Trauma causes significant global burden of disease. Trauma life support training programmes aim to improve early trauma care, but little is known about their effects on patient outcomes.

**Methods:**

We conducted a systematic review using keywords and medical subject headings (MeSH-terms) related to trauma life support training in seven online databases: Medline, Embase, Cochrane, Web of Science, Global Health, CINAHL and Google Scholar. The reference lists of included articles were also searched for relevant studies. Published studies that compared patient outcomes between providers trained in any trauma life support training programme with those trained in another training programme or those not trained were included. We appraised the quality of the included studies and conducted meta-analyses using random effects models.

**Results:**

We screened 9,626 records from which we identified and included 19 eligible studies. There were 92,614 patients cumulatively across the 19 studies and Advanced Trauma Life Support (ATLS) was the most common trauma life support training programme. Seventeen of the studies were included in a meta-analysis with mortality as the outcome. Trauma life support training programmes were associated with reduced mortality at an odds ratio of 0.60 (95% CI 0.48–0.75). The total heterogeneity (I^2^) was 74.4% and the publication bias for the Egger’s regression test was p = 0.2, and the Rank correlation test p = 0.90.

**Conclusions:**

Trauma life support training programmes are associated with reduced mortality in trauma patients, but the evidence is observational. Future research should therefore focus on the effects of these training programmes on patient outcomes using randomised controlled or high-quality quasi-experimental designs.

**Supplementary Information:**

The online version contains supplementary material available at 10.1186/s13049-026-01549-w.

## Introduction

Trauma accounts for the deaths of 4.4 million people each year, constituting 8% of all mortality and is the sixth highest cause of disability-adjusted life years globally [1, 2]. Up to 42% of patients experience various forms of disability post-trauma, impacting their ability to work, limiting their socioeconomic opportunities, and potentially leading to poverty [3]. These adverse effects extend to the economy, with the global cost of acute treatment amounting to approximately 518 billion USD each year [4].

Trauma life support training programmes such as the Advanced Trauma Life Support (ATLS) programme, aim to enhance the knowledge and skills of the providers leading to changes in behaviour and practice that can result in better patient outcomes and have been used to train over one million healthcare providers in over 80 countries [5, 6]. Several studies have shown that training improves the skills of healthcare providers by improving their confidence, cognitive and clinical skills, and knowledge in managing patients [7–9].

Previous systematic reviews have been conducted to establish the effects of trauma life support training on patient and provider outcomes [8, 10–14]. However, these reviews focus on specific programmes [8, 10, 12, 14], country income levels [11], or have primarily evaluated the improvements in the providers’ skills [8, 12, 13]. Without understanding the effects of trauma life support training on patient outcomes, healthcare systems lack a comparative basis for implementing such programmes.

Our systematic review, therefore, aims to assess the effects of trauma life support training programmes on patient outcomes.

## Method

We formulated the question for this systematic review using the Population, Intervention, Comparison, and Outcome (PICO) framework. The population of interest was trauma patients, the intervention was any trauma life support training programme, the comparator was “no training”, or alternative training programmes, and the outcomes assessed were mortality, morbidity, and return to work. 

This study is reported using the Preferred Reporting Items for Systematic Reviews and Meta-Analyses (PRISMA) guidelines, and the protocol was registered in PROSPERO (CRD42022373977) before the commencement of the study [15, 16].

### Protocol deviations

Deviations from the original protocol on PROSPERO included the extension of databases searched, from four to seven, for enhanced validity. The references of the selected articles were also appraised as part of the search strategy, and trial registers were excluded as the focus was on peer-reviewed studies. The assessment of the main outcomes, particularly the precise time at which mortality was measured, could not be conducted because this information was not provided in most of the articles.

### Eligibility criteria

Peer-reviewed studies on the effects of any trauma life support training programme of hospital healthcare providers on trauma patient outcomes were eligible.

#### Inclusion criteria

Articles reporting studies that compared the outcomes of patients treated in-hospital by healthcare providers trained in a trauma life support training programme to those trained in a different trauma life support programme, or those who were not trained. The study had to be conducted in a hospital, and the healthcare providers who underwent training had to be certified medical staff. Articles could be controlled trials as well as prospective or retrospective observational studies.

#### Exclusion criteria

Articles whose main text was not in English were not used, as the team’s working language was English. Articles that were reviews, where the full text was not available, studies where only a sub-group of the population was being studied (e.g., geriatric patients), those where pre-hospital staff were trained, and studies where outcomes were measured before patients arrived at the hospital, were also excluded.

### Information sources

The literature search was conducted using seven online databases: Medline, Embase, Cochrane, Web of Science, Global Health, CINAHL, and Google Scholar. The search period for each was from the inception of each database until the 11th of August 2025. The reference lists of the selected articles were also searched on the 08th of September 2025.

### Search strategy

The search strategy, which was developed in collaboration with the Karolinska Institutet’s University Library, utilised Medical Subject Headings (MeSH terms) and free-text term for each search concept (Supplementary Tables 1–7). The strategy was initially deployed in Medline, the primary database and then translated into the six other databases. The resulting records were de-duplicated using the Bramer et al. method [17] with the additional step of comparing the digital object identifiers (DOIs) in EndNote [18].

### Study selection

Following the search, the results were imported into Covidence [19], where they were screened by at least two reviewers independently, with a third reviewer being brought in for consensus. First, the records were screened based on their titles and abstracts, and then the selected records were further screened based on their full-text articles. Full-text articles that were behind a paywall were first searched for through the Karolinska Institutet’s library subscription with publishers and then were requested through direct contact with the authors.

### Data collection process and data items

The data from the resulting articles were independently extracted by at least two reviewers, with a discussion being held to reach consensus. The data collection tool was customised in Covidence, and the extraction characteristics were study type, country, urban/rural location, the type of hospital, trauma training programme, outcomes studied, staff characteristics, patient characteristics, injury severity, average age, sex, and sample size. Outcome time frames were extracted as reported. Studies that only reported in-hospital mortality without a specific time frame were grouped under an “in-hospital” category. The outcome measures extracted included the pre-training or control summary measure of the outcome, the post-training or intervention summary measure and the differences between these measures, as well as any associated confidence intervals. Finally, any information on ethical considerations taken, such as obtaining patient consent, board approval, and declaring any funding and/or conflicting interests, was recorded.

### Study risk of bias assessment

Two reviewers independently conducted the study risk of bias assessment using the Scottish Intercollegiate Guidelines Network (SIGN) checklist for cohort studies as all included studies were either prospective or retrospective cohort studies [20]. The studies were rated as either unacceptable (-), acceptable (+), or high quality (++) depending on a series of questions rating how well the study had done to minimise the risk of bias or confounding. A discussion was then held to reach consensus on the score for the papers.

### Meta-analysis

Studies that had a SIGN score of acceptable or above and those that measured the same outcome within a comparable time frame were included in a meta-analysis, which was conducted using R statistical software with the “metafor” package [21, 22]. The I^2^ statistic was determined, and a random effects model was used to calculate the odds ratio (OR) of the outcome. The pooled effects size was visualised using a Forest plot at a 95% confidence interval (CI). To assess the publication bias, a funnel plot, Egger’s Regression Test and rank test were performed.

We conducted multiple sensitivity analyses. First, a leave-one-out analysis was performed to assess the effects of excluding one study at a time from the main analysis. Second, we conducted sensitivity meta-analyses according to the trauma life support training programme studied, the outcome definition used, and the income level of the country where the study took place, using the World Bank’s country income level definitions [23].

### Ethical considerations

Since no patient details were used for the systematic review, ethical approval was not required. However, ethical considerations of the included studies were recorded.

## Results

### Study selection

**Fig. 1:**
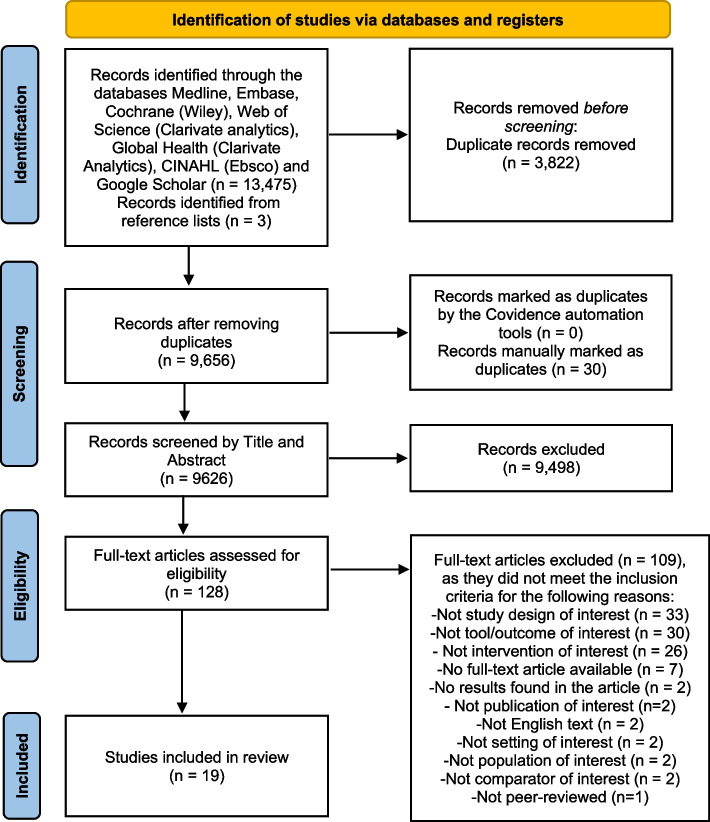
Selection of studies according to the PRISMA flowchart

The search results yielded 13,475 records, of which 3,852 were identified as duplicates and removed. Only three records were found through the reference lists. 9,626 records were screened in the title and abstract round, from which 128 were assessed for eligibility in the full-text assessment. 19 studies were then included in the final extraction round, as seen in Fig. 1.

### Study characteristics

The characteristics of the 19 studies included in this review are described in Table 1. In these 19 studies, most of the participants were male, 35 to 48 years of age. The studies were all cohort studies, eight of which were prospective [24–31] and eleven of which were retrospective [32–42]. They were set in high-income [24, 25, 28, 30, 33, 34, 39, 40, 42], upper-middle-income [29, 32, 35–37], lower-middle-income [26, 31, 38, 41] and low-income countries [27]. The majority were conducted in urban areas [24–29, 31–34, 36–39, 41]. Eleven studies assessed ATLS [24–26, 28, 34–39, 41], two assessed PTC [29, 31], and six looked at rural, custom courses or trauma team training courses [27, 30, 32, 33, 40, 42]. None of the studies compared two trauma life support training programmes. As shown in Table 2, all of the studies assessed mortality as the outcome [24–42].

**Table 1:** Descriptive summary of the 16 included studies

Study	Study Type	Setting	Course	SIGN Risk of Bias
Vestrup 1988 [34]	Retrospective cohort	Canada, HIC, Urban, Public hospital- Level 1 trauma centre	ATLS	Acceptable (+)
Ariyanayagam 1992 [35]	Retrospective cohort	Trinidad and Tobago, UMIC*, Hospital	ATLS	Unacceptable—reject 0
Ali 1993 [36]	Retrospective cohort	Trinidad and Tobago, UMIC*, Urban, Public hospital- major trauma referral hospital	ATLS	Acceptable (+)
van Olden 2004 [24]	Prospective, sequential, cohort	The Netherlands, HIC, Urban, ACS level III community residency training hospitals	ATLS	Acceptable (+)
Wang 2010 [37]	Retrospective cohort	China, UMIC, Urban, Public hospital	ATLS	Acceptable (+)
Drimousis 2011 [25]	Prospective observational	Greece, HIC, Urban and Rural, Public hospitals- mainly primary and secondary healthcare centres	ATLS	Unacceptable—reject 0
Noordin 2011 [26]	Prospective study retrospectively analysed	Pakistan, LMIC, Urban, Private university hospital	ATLS	High Quality (++)
Hashmi 2013 [38]	Retrospective cohort	Pakistan, LMIC, Urban, Private university hospital- primary and referral trauma centre	ATLS (protocols as part of trauma improvement quality (TIQ) implementation)	High Quality (++)
Hondo 2013 [33]	Retrospective cohort	Japan, HIC, Urban and Rural, Mostly private hospitals across Japan	JATEC	Acceptable (+)
Petroze 2015 [27]	Prospective cohort	Rwanda, LIC, Urban, Public university hospital	ATLS (provider demonstration, Canadian Network for International Surgery), TTT	Acceptable (+)
Bellanova 2016 [28]	Prospective cohort	Italy, HIC, Urban, Public provincial hospital	ATLS, E-FAST, Clinical Audit "Thursday of Trauma", Management of Politrauma	Acceptable (+)
Dennis 2016 [40]	Retrospective cohort	USA, HIC, Rural, Rural non-trauma hospitals, private hospital—level 1 trauma centre	RTTDC	Acceptable (+)
Magnone 2016 [39]	Retrospective cohort	Italy, HIC, Urban, Semi-private metropolitan hospital	ATLS	Acceptable (+)
Cioè-Peña 2016 [29]	Prospective cohort	El Salvador, UMIC, Urban, Hospital	PTC	Acceptable (+)
Yao 2018 [32]	Retrospective cohort	China, UMIC, Urban, Public hospital and a provincial government owned trauma medical centre	CTCT	Acceptable (+)
Bauman 2024 [30]	Prospective cohort	USA, HIC, Rural, Level 1 academic trauma hospital	RTTDC	High Quality (++)
Kamau 2024 [41]	Retrospective cohort	Kenya, LMIC, Urban, Private hospital	ATLS	Acceptable (+)
Nguyen 2025 [31]	Prospective Cohort	Viet Nam, LMIC, Urban, Trauma centre	PTC	Acceptable (+)
Priestap 2025 [42]	Retrospective cohort	Canada, HIC, Mixed (rural with urban leadership), 6 referral hospitals and 1 lead trauma hospital	RTTDC	Acceptable (+)

Abbreviations: ATLS- Advanced Trauma Life Support Training, CTCT- China Trauma Care Training, E-FAST- Extended Focused Assessment with Sonography for Trauma, HIC- High-income country, JATEC-Japan Advanced Trauma Education and Care, LIC- Low-income country, LMIC- Lower-middle income country, PTC- Primary Trauma Care, RTTDC- Rural Trauma Team Development Course, TTT- Trauma Team Training, UMIC- Upper-middle income country

^*^Trinidad and Tobago was classified by the World Bank as an upper-middle-income country in 1992 and 1993 [23]

**Table 2:** Statistical summary of the effect of trauma life support training on patient outcomes

Study	Outcome	Time of measurement	Total sample size	Pre-intervention/Control sample size	Post-intervention/Intervention sample size	Pre-intervention/Control events, n (%)	Post-intervention/Intervention events, n (%)
Vestrup 1988 [34]	Mortality	In-hospital	121	50	71	13 (26)	14 (20)
Ariyanayagam 1992 [35]	Mortality	6 h	22,871	13739^a^	9132^a^	302 (2.2)^a^	192 (2.1)^a^
		In-hospital				637 (4.6)^a^	430 (4.7)^a^
Ali 1993 [36]	Mortality	In hospital	813	413	400	279 (67.6)	134 (33.5)
	Minor disabilities^b^	3 months	400	134	266	30 (22.4)	235 (88.3)
	Moderate disabilities^b^					95 (70.9)	26 (9.8)
	Major disabilities^b^					9 (9.8)	5 (1.9)
van Olden 2004 [24]	Mortality	Total mortality (< 1 h, 1 day, weeks)	63	31	32	15 (48)	10 (31)
	Morbidity	Not reported				-	-
Wang 2010 [37]	Mortality	In-hospital	820	438	382	87 (19.9)	62 (15.1)
Drimousis 2011 [25]	Mortality	In-hospital	8862	6734	1431	128 (2)	53 (3.7)
Noordin 2011 [26]	Mortality	In-hospital	1009	435	574	42 (9.7)	33 (5.7)
Hashmi 2013 [38]	Mortality	In-hospital	1227	421	806	40 (9.8)	39 (4.9)
Hondo 2013 [33]	Mortality	In-hospital	47,095	6495	27,787	864 (13.3)^c^	2362 (8.5)^c^
Petroze 2015 [27]	Mortality	30 days	1373	798	575	96 (12)	59 (10.3)
Bellanova 2016 [28]	Mortality	48 h	230	98	132	9 (9)	5 (4.5)
Dennis 2016 [40]	Mortality	In-hospital	253	61	69	1 (2)	5 (7)
Magnone 2016 [39]	Mortality	24 h	339	198	141	28 (14.1)	10 (7.1)
Cioè-Peña 2016 [29]	Mortality	In hospital	194	48	146	5 (10)	18 (12)
Yao 2018 [32]	Mortality	In-hospital	840	404	436	76 (18.8)	67 (15.4)
Bauman 2024 [30]	Mortality	In-hospital	472	240	232	10 (4.2)	10 (4.3)
Kamau 2024 [41]	Mortality	30 days	162	81	81	14 (17)	5 (6)
Nguyen 2025 [31]	Mortality	24 h	5690	2965	2725	87 (2.9)	28 (1)
		30 days	3630	2031	1599	97 (4.6)	39 (2.4)
Priestap 2025 [42]	Mortality	In-hospital	180	90	90	6 (6.7)	7 (7.8)

^a^ The values for the pre- and post-training population samples could not be derived using the data from the original article. Several attempts to contact the authors also proved unsuccessful. The results in this table were taken from Putra et al. 2023 [14]

^b^Ali et al. [36] defined minor disabilities as a patient returning to the same level of employment, moderate disabilities as a patient returning to a lower level of employment based on physical activity, and major disabilities as a patient discharged home but unemployable, vegetative or chronically institutionalised

^c^ Hondo et al. [33] measured mortality during the early training period (before training was implemented), during the transition period (while training was ongoing) and during the late training period (after training was implemented). For the purposes of this study, only the outcomes for the early training period and the late training period were used

### Risk of bias in studies

Three of the 19 studies were assessed as being high quality (++), due to how well they minimised the risk of bias or confounding. Fourteen of the studies were only rated as acceptable (+) because the proper statistics on the patient population, e.g., the age of the sample population or the male-to-female ratio, were not given, thereby introducing information bias. Two of the studies were rated unacceptable because they contained information biases as well as no mention of possible confounders (Table 1).

### Results of individual studies

Seventeen of the studies passed the quality assessment and were included in the meta-analysis [24, 26–34, 36–42].

#### Mortality

**Fig. 2:**
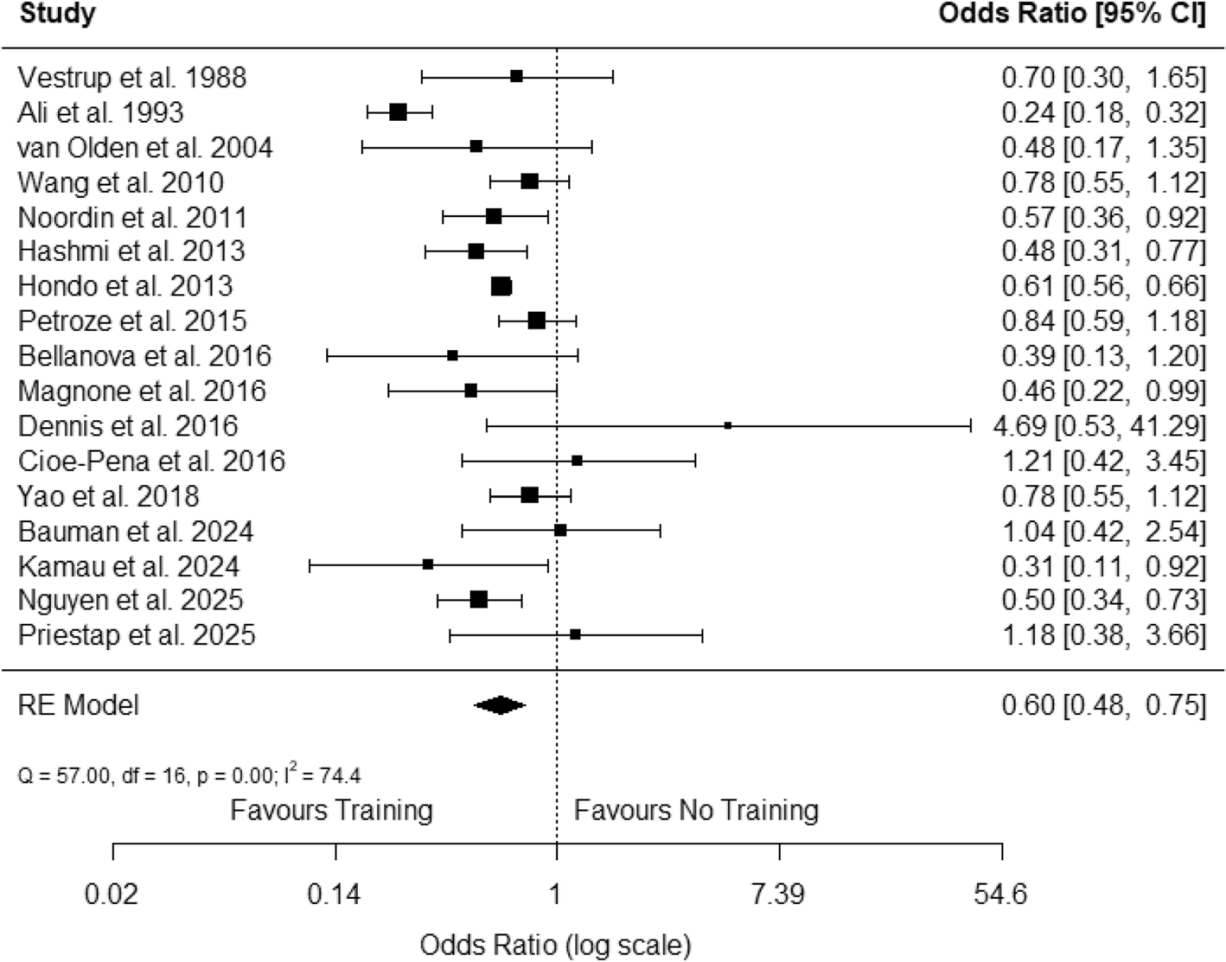
Forest plot of individual and pooled odds ratio for mortality across 17 included studies. For Nguyen et al. [31], the 30-day mortality estimate was included in this main meta-analysis. Abbreviations: RE- Random Effects

The weighted pooled OR comparing the effect of “trauma life support training” with “no training” on mortality across the 17 studies was 0.60 (95% CI 0.48—0.75) as shown in Fig. 2, indicating that, on average, the implementation of in-hospital trauma life support training programmes reduced the odds of mortality by 40%.

The p-value was < 0.05 and the I^2^ statistic showed a 74.4% heterogeneity between the studies (Supplementary Table 8), thereby representing substantial heterogeneity. The Tau^2^ estimation was 0.12 (95% CI 0.03—0.53) with a standard error (SE) of 0.07 (Supplementary Table 8).

#### Morbidity and return to work

Ali et al. was the only study reporting on morbidity as a combination of return to work and disability [36]. They found that moderate and major disabilities were substantially reduced after the implementation of ATLS (Table 2) [36].

#### Publication bias

**Fig. 3:**
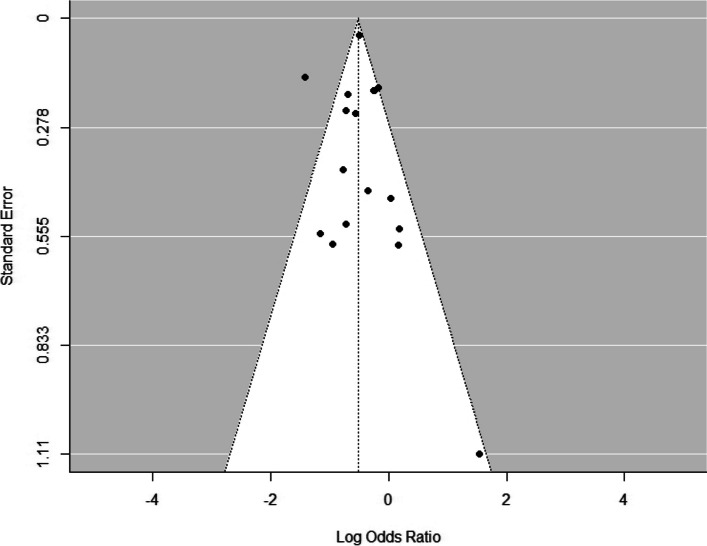
A funnel plot displaying the publication bias of the studies. *NB: The Wang et al. [37] and Yao et al. [32] studies overlap, which is why only 16 dots are visible

The publication bias was assessed using a Funnel plot as shown in Fig. 3, and the Egger’s regression test and rank correlation test as shown in Supplementary Table 8. These results indicate that there was not much publication bias present.

### Sensitivity analysis

The leave-one-out analysis (see Supplementary Table 9) indicates that the high statistical heterogeneity across the studies (I^2^ = 74.4%) was primarily attributable to the study by Ali et al. [36], which reported a comparatively large effect size and smaller variance in its estimate relative to the other studies. A second meta-analysis was therefore conducted without the Ali et al. [36] study, which revealed a similar effect on mortality (weighted-pooled OR 0.64, (95% CI 0.57—0.72)), but with a substantially lower statistical heterogeneity (I^2^ = 14.4%, *p*-value < 0.05).

**Fig. 4:**
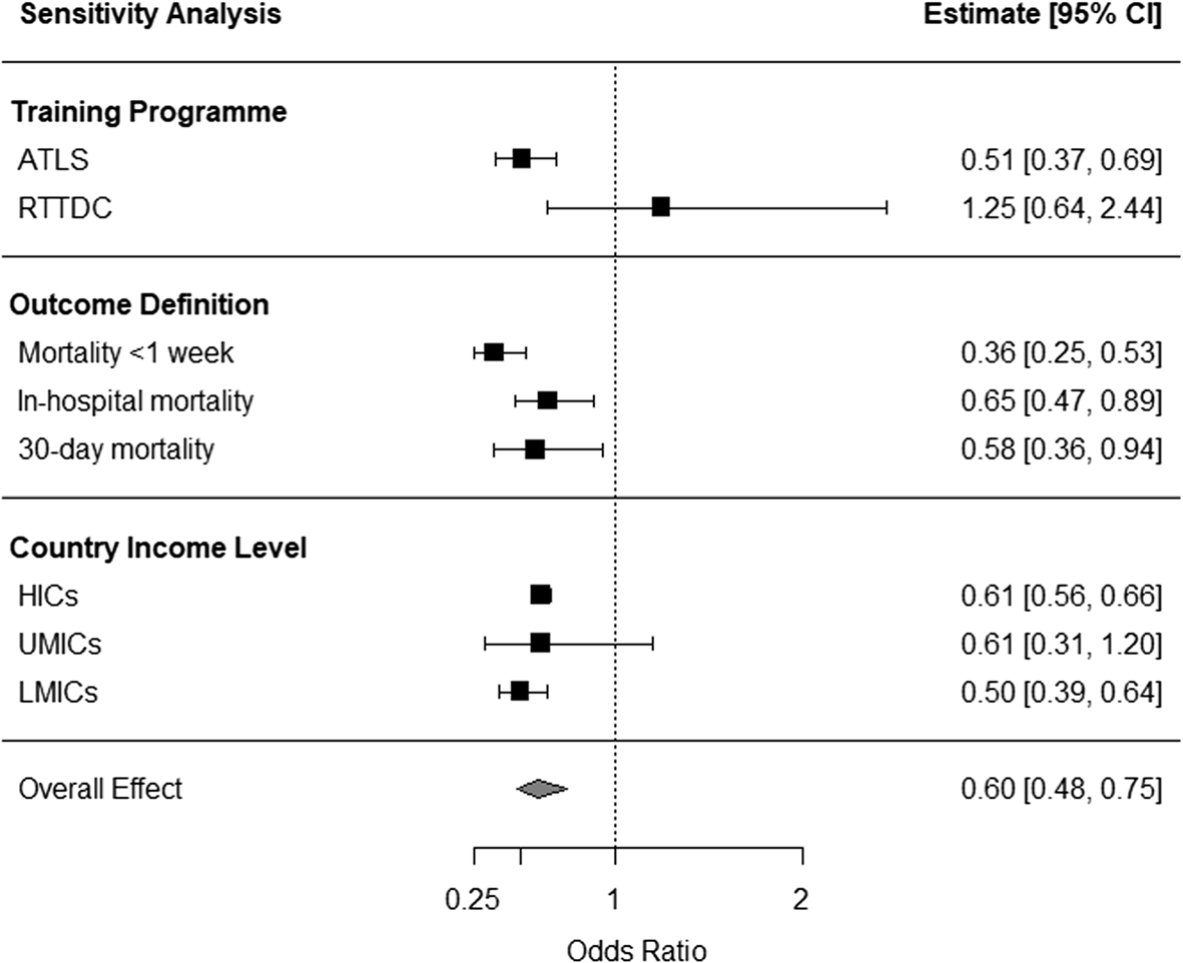
Forest plot summarising the weighted pooled odds ratio and confidence intervals of the sensitivity meta-analyses. Full results are available as supplementary material. Abbreviations: ATLS- Advanced Trauma Life Support, HICs- High-income countries, LMICs- Lower-middle-income countries, RTTDC- Rural Trauma Team Development Course, UMICs- Upper-middle-income countries

Further sensitivity analyses were conducted according to the specific trauma life support training programme, mortality outcome definition, and country income level (shown in Fig. 4), when there were more than two studies available for each analysis. Across sensitivity analyses, trauma life support training was associated with reduced mortality, with the exception of RTTDC, which was associated with a non-significant increase in mortality. Detailed results for all sensitivity analyses are available in the supplementary materials.

### Ethical considerations of the included studies

An appraisal of the ethical steps mentioned by the individual studies was done, which revealed that only seven studies [26, 29–31, 40–42] mentioned receiving board approval, but only three took steps to ensure patient confidentiality [26, 29, 41]. Four of the studies did not declare whether they had conflicts of interest, had received funding, or had received board approval [28, 33, 35, 36]. Eight papers did not mention any steps taken to protect patient information, but five of those were retrospective, and the other three either mentioned their conflicts of interest or whether funding was received [24, 25, 27, 32, 34, 37–39].

## Discussion

Our study indicates that trauma life support training programmes are associated with reduced mortality among trauma patients. Similar conclusions regarding morbidity and return to work could not be made due to limited data. Our findings are consistent with previous systematic reviews by Jabbour et al. and Jin et al., which also reported significant reductions in mortality [11, 13], while reviews by Noonan et al. and Putra et al. reported similar point estimates but did not find any significant effects on mortality [12, 14].

Jabbour et al. [13] assessed ATLS as an intervention using 17 articles, but only three assessed mortality and were used in their meta-analysis. They reported a pooled OR of 0.28 (95% CI 0.22-0.37), which is substantially lower than the pooled OR of our study. Jin et al. assessed in-hospital trauma training in low- and middle-income countries and reported a pooled risk ratio of 0.55 (95% CI 0.4–0.75) based on seven studies [11], which is consistent with our sensitivity analyses of studies conducted in lower-middle-income countries. Noonan et al. assessed the effect of trauma team training and reported a pooled OR of 0.83 (95% CI, 0.64–1.09), using seven articles in their meta-analysis [12]. Putra et al. studied ATLS and reported a pooled OR of 0.68 (95% CI 0.39–1.20), also using seven studies [14].

Our pooled results contrast with several individual papers. The studies by Drimousis et al. and Cioè-Peña et al. both found increased mortality after ATLS and PTC training, respectively [25, 29], while the three studies that investigated the Rural Trauma Team Development Course (RTTDC) failed to show a decrease in mortality post-training [30, 40, 42]. This is in contrast to a recently conducted cluster randomised trial by Lule et al. [43], which found that RTTDC significantly reduced mortality among patients injured in motorcycle crashes. However, as the paper is still only available as a preprint, it did not meet our inclusion criteria.

The results of our meta-analysis revealed high statistical heterogeneity (I^2^ = 74.4%) in the primary pooled estimate, which was largely driven by the Ali et al. study [36] according to the leave-one-out analysis. By removing this study, the heterogeneity reduced to 14.4% while the pooled OR remained relatively the same. This indicates that the overall finding of an association with reduced mortality was not dependent on a single study. Additionally, to account for the high heterogeneity, we used random effects models and performed several sensitivity analyses to examine the robustness of our results.

The substantial statistical heterogeneity in our pooled results likely reflects clinical and methodological differences among the included studies, which differed in terms of the programme being studied, its duration, delivery, setting, and time point when the outcomes were measured. In addition to these reported differences, some articles excluded important clinical information such as the injury severity, age distribution, sex ratio and sample size, and many did not specify the time point when the outcome was measured. These differences between the included studies likely influenced the reported effect sizes.

We decided to pool results across studies in our main analysis despite these differences. Our main rationale for doing so, was that even if these implementation characteristics differed among these specific studies, all trauma life support training programmes share the same basic principles and have the common goal of improving the skills of trauma care providers and ultimately patient outcomes. The main pooled estimate is therefore useful because it provides an indication of the direction of the overall effect.

Our decision to pool results across studies does affect the interpretation of the pooled estimate, especially with regards to the different time-points when mortality was measured. Early and late mortality reflect different underlying mechanisms such as early mortality due to severity of injuries versus later mortality due to complications. As is evident from the sensitivity analysis, studies on early mortality are more likely to show a large effect, thereby exaggerating the pooled effect size.

Notwithstanding the heterogeneity, the main analysis and the majority of sensitivity analyses indicate that trauma life support training is associated with reduced mortality, even if strong evidence from randomised or high-quality quasi-experimental studies is still lacking. It is beyond the scope of this study to propose the mechanism through which trauma life support training may work to improve patient outcomes, as this is likely to result from a complex interplay between outer and internal settings, the individuals trained and the patients they manage and is unlikely to happen in the absence of system-level improvement. However, Mohammad et al. compiled level 1 and 2 evidence showing that ATLS training improves providers’ organisational, priority setting, and clinical skills in treating trauma patients [8], which are likely to be necessary mediators of the relationship between training and patient outcomes.

There are several potential biases that may have influenced our findings. First, our review included only peer-reviewed articles from just seven databases and was limited to English-language studies due to this being the primary language of the research team, and relevant articles in other languages might have been missed. In addition, several articles did not disclose conflicts of interest or funding sources, raising the possibility of sponsor influence. Although most studies were retrospective, Institutional Review Board approval remains important for compliance with research regulations and international standards, yet it was often not reported [44]. Finally, while our results indicate the effectiveness of trauma life support training in high-income and middle-income settings, generalisation to low-income and rural areas is limited.

Future research should most importantly, strive to use randomised or high-quality quasi-experimental designs. These studies should also measure mortality at standardised time points, evaluate the impact of these training programmes on morbidity and return to work, and report patient injury severity as well as pre-hospital and in-hospital care conditions, as these factors that can influence the training programme’s effectiveness.

To our knowledge, this is the first systematic review that attempted to assess the effects of any trauma life support training programme across country income levels on patient outcomes. In the absence of higher-quality evidence, our study supports the use of trauma life support training across settings. Our analysis also identified gaps in the literature, particularly the absence of high-quality controlled research as well as research on the impact of these training programmes on morbidity and return to work.

## Conclusion

Observational research indicates that trauma life support training is associated with reduced mortality, but high-quality evidence from randomised or high-quality quasi-experimental studies is lacking. The effects of trauma life support training on morbidity and return to work cannot be established.

## Supplementary Information

Supplementary Material 1.

## Data Availability

The materials and data are available upon request.

## Abbreviations

ATLS: Advanced Trauma Life Support
CI: Confidence interval
CTCT: China Trauma Care Training
DALYs: Disability-adjusted life years
E-FAST: Extended Focused Assessment with Sonography for Trauma
DOIs: Digital Object Identifiers
HICs: High-income countries
IRB: Institutional Review Board
ISS: Injury Severity Score
JATEC: Japan Advanced Trauma Education and Care
LICs: Low-income countries
LMICs: Low- and middle-income countries
MeSH: Medical Subject Headings
OR: Odds ratio
PRISMA: Preferred Reporting Items for Systematic Reviews and Meta-Analyses
PTC: Primary Trauma Care
RR: Risk ratio
RTTDC: Rural Trauma Team Development Course
SIGN: Scottish Intercollegiate Guidelines Network
TTT: Trauma Team Training
UMICs: Upper-middle-income countries
WHO: World Health Organisation
