# Supplementary material for: Effect of trauma life support training on patient outcomes: a systematic review and meta-analysis
Source: Scand J Trauma Resusc Emerg Med. 2026 Jan 20;34:15. doi: 10.1186/s13049-026-01549-w (PMC12849152; doi:10.1186/s13049-026-01549-w)
Supplement: Supplementary file 1 — Supplementary Material 1. [file 13049_2026_1549_MOESM1_ESM.pdf]

Supplementary Materials

Effect of Trauma Life Support Training on Patient Outcomes: A Systematic Review and Meta-analysis

Authors

Zaynab Nakhid[1] MMSc.

Martin Gerdin Wärnberg[1,2] MD, PhD

Johanna Berg,[1,3] MD

Kapil Dev Soni[4] MD, MS

Monty Khajanchi[5] MD, MS

Deepa Kizhakke Veetil[6] MD, MS

Siddarth Daniels David[1] PhD

Affiliations

1. Department of Global Public Health, Karolinska Institutet, Stockholm, Sweden.
2. Function Perioperative Medicine and Intensive Care, Karolinska University Hospital, Solna, Sweden.
3. Department of Emergency Medicine, Skåne University Hospital, Malmö, Sweden.
4. JPN Apex Trauma Center All India Institute of Medical Sciences, New Delhi, India.
5. GSMC & KEM Hospital Mumbai, India. WHOCC for Research in Surgical Care Delivery in LMICs.
6. Department of Minimal Access Surgery Manipal Hospitals, Dwarka, Delhi, India.

Corresponding author

Siddarth Daniels David, +91 98197 40616, siddarth.david@ki.se,

Supplementary Materials Section 1:

Supplementary Table 1. Medline Search Strategy

Interface: Ovid MEDLINE(R) and Epub Ahead of Print, In-Process & Other Non-Indexed Citations and Daily<br><br>Date of Search: 11 August 2025<br><br>Number of hits: 3,176<br><br>Comment: In Ovid, two or more words are automatically searched as phrases; i.e. no quotation marks are needed	Field labels<br>• exp/ = exploded MeSH term<br>• / = non exploded MeSH term<br>• .ti,ab,kf. = title, abstract and author keywords<br>• adjx = within x words, regardless of order<br>• * = truncation of word for alternate endings

Database(s): **Ovid MEDLINE(R) ALL** 1946 to August 08, 2025
Search Strategy:

#	Searches	Results
1	Traumatology/	3979
2	Trauma Centers/	14618
3	Multiple Trauma/	13862
4	"Wounds and Injuries"/th	17419
5	Advanced Trauma Life Support Care/	206
6	or/1-5	45427
7	education.fs.	319104
8	Education, Medical, Continuing/	25797
9	Educational Measurement/	44331
10	Education, Medical/	63162
11	Education, Medical, Undergraduate/	29483
12	Inservice Training/	20860
13	Education, Professional/	3126
14	Educational Status/	63618
15	or/7-14	477512
16	6 and 15	2238
17	((ATAM or ATLS or PTC or PHTLS or trauma or trauma care or trauma team or trauma team development or trauma management or trauma training) adj (course or continuing education or continuing professional development or curriculum? or education* or program* or training* or workshop?)).ti,ab,kf.	1133

18	(trauma adj3 (continuing education or education* course? or education* program* or training course? or training program*)).ti,ab,kf.	228
19	((trauma care or trauma team?) and (continuing education or education* course? or education* program* or training course? or training program*)).ti,ab,kf.	281
20	((advanced trauma life support or primary trauma care or "trauma assessment and management" or "trauma evaluation and management" or prehospital trauma life support) and (course? or curriculum? or education* or program* or training* or workshop?)).ti,ab,kf.	517
21	or/17-20	1678
22	16 or 21	3478
**23**	**limit 22 to english**	**3176**

Supplementary Table 2. Embase Search Strategy

Interface: embase.com<br>Date of Search: 11 August 2025<br>Number of hits: 6,359<br>Comment: Emtree is the controlled vocabulary in Embase	Field labels<br>• /exp = exploded Emtree term<br>• /de = non exploded Emtree term<br>• ti,ab,kw = title, abstract and author keywords<br>• NEAR/x = within x words, regardless of order<br>• * = truncation of word for alternate endings

#	Searches	Results
#01	'traumatology'/exp	13557
#02	'multiple trauma'/de	18332
#03	'injury'/mj	92574
#04	'emergency care'/exp	67045
#05	#1 OR #2 OR #3 OR #4	186952
#06	'medical education'/de OR 'residency education'/de	314202
#07	'education'/de OR 'education program'/de	587172
#08	'in service training'/de	16944
#09	'vocational education'/de OR 'continuing education'/de	47237
#10	'paramedical education'/de	4044
#11	'staff training'/de	17349
#12	'course evaluation'/de OR 'course content'/de	4011
#13	'curriculum development'/de	6130
#14	#6 OR #7 OR #8 OR #9 OR #10 OR #11 OR #12 OR #13	880035
#15	#5 AND #14	9580
#16	((atam OR atls OR ptc OR phtls OR trauma OR 'trauma care' OR 'trauma team' OR 'trauma team development' OR 'trauma management' OR 'trauma training') NEXT/1 (course$ OR 'continuing education' OR 'continuing professional development' OR curriculum$ OR education* OR program* OR training* OR workshop$)):ti,ab,kw	1721
#17	(trauma NEAR/3 ('continuing education' OR 'education* course$' OR 'education* program*' OR 'training course$' OR 'training program*')):ti,ab,kw	285
#18	('trauma care':ti,ab,kw OR 'trauma team$':ti,ab,kw) AND ('continuing education':ti,ab,kw OR 'education* course$':ti,ab,kw OR 'education* program*':ti,ab,kw OR 'training course$':ti,ab,kw OR 'training program*':ti,ab,kw)	359
#19	('advanced trauma life support':ti,ab,kw OR 'primary trauma care':ti,ab,kw OR 'trauma assessment and management':ti,ab,kw OR 'trauma evaluation and management':ti,ab,kw OR 'prehospital trauma life support':ti,ab,kw) AND (course$:ti,ab,kw OR	641

	curriculum$:ti,ab,kw OR education*:ti,ab,kw OR program*:ti,ab,kw OR training*:ti,ab,kw OR workshop$:ti,ab,kw)	
#20	#16 OR #17 OR #18 OR #19	2306
#21	#15 OR #20	11311
#22	(#15 OR #20) AND [english]/lim	10531
**#23**	**#22 AND ('article'/it OR 'article in press'/it OR 'erratum'/it OR 'preprint'/it OR 'review'/it OR 'short survey'/it OR 'conference paper'/it)**	**6359**

Supplementary Table 3. Wiley Search Strategy

Interface: Wiley<br>Date of Search: 11 August 2025<br>Number of hits: 121	Field labels<br>• ti,ab,kw = title, abstract and author keywords<br>• NEAR/x = within x words, regardless of order<br>• * = truncation of word for alternate endings

#	Searches	Results
#1	((ATAM:ti,ab,kw OR ATLS:ti,ab,kw OR PTC:ti,ab,kw OR PHTLS:ti,ab,kw OR trauma:ti,ab,kw OR "trauma care":ti,ab,kw OR "trauma team":ti,ab,kw OR "trauma team development":ti,ab,kw OR "trauma management":ti,ab,kw OR "trauma training":ti,ab,kw) NEXT (course*:ti,ab,kw OR "continuing education":ti,ab,kw OR "continuing professional development":ti,ab,kw OR curriculum*:ti,ab,kw OR education*:ti,ab,kw OR program*:ti,ab,kw OR training*:ti,ab,kw OR workshop*:ti,ab,kw))	86
#2	(trauma:ti,ab,kw NEAR/3 ("continuing education":ti,ab,kw OR (education* NEXT course*):ti,ab,kw OR (education* NEXT program*):ti,ab,kw OR ("training" NEXT course*):ti,ab,kw OR ("training" NEXT program*):ti,ab,kw))	19
#3	(("trauma care":ti,ab,kw OR ("trauma" NEXT team*):ti,ab,kw) AND ("continuing education":ti,ab,kw OR (education* NEXT course*):ti,ab,kw OR (education* NEXT program*):ti,ab,kw OR ("training" NEXT course*):ti,ab,kw OR ("training" NEXT program*):ti,ab,kw))	16
#4	(("advanced trauma life support":ti,ab,kw OR "primary trauma care":ti,ab,kw OR "trauma assessment and management":ti,ab,kw OR "trauma evaluation and management":ti,ab,kw OR "prehospital trauma life support":ti,ab,kw) AND (course*:ti,ab,kw OR curriculum*:ti,ab,kw OR education*:ti,ab,kw OR program*:ti,ab,kw OR training*:ti,ab,kw OR workshop*:ti,ab,kw))	48
**#5**	**#1 OR #2 OR #3 OR #4**	**121**

Supplementary Table 4. Web of Science Core Collection Search Strategy

Interface: Clarivate Analytics<br><br>Editions = A&HCI , ESCI , SCI-EXPANDED , SSCI<br><br>Date of Search: 11 August 2025<br><br>Number of hits: 1,915	Field labels<br><br>• TS/Topic = title, abstract, author keywords and Keywords Plus<br>• NEAR/x = within x words, regardless of order<br>• * = truncation of word for alternate endings<br><br>Note: the *Exact search*-function was used for all the searches

#	Search Query	Results
1	TS=((ATAM OR ATLS OR PTC OR PHTLS OR trauma OR "trauma care" OR "trauma team" OR "trauma team development" OR "trauma management" OR "trauma training") NEAR/0 (course$ OR "continuing education" OR "continuing professional development" OR curriculum$ OR education* OR program* OR training* OR workshop$))	1469
2	TS=(trauma NEAR/3 ("continuing education" OR "education* course$" OR "education* program*" OR "training course$" OR "training program*"))	307
3	TS=(("trauma care" OR "trauma team$") AND ("continuing education" OR "education* course$" OR "education* program*" OR "training course$" OR "training program*"))	300
4	TS=(("advanced trauma life support" OR "primary trauma care" OR "trauma assessment and management" OR "trauma evaluation and management" OR "prehospital trauma life support") AND (course$ OR curriculum$ OR education* OR program* OR training* OR workshop$))	487
5	#4 OR #3 OR #2 OR #1	2013
**6**	**#4 OR #3 OR #2 OR #1 and English (languages)**	**1915**

Supplementary Table 5. Global Health Search Strategy

Interface: Clarivate Analytics<br><br>CABI: Global Health® (1984-present)<br><br>Date of Search: 11 August 2025<br><br>Number of hits: 144	Field labels<br>• TS/Topic = title, abstract, author keywords and Keywords Plus<br>• NEAR/x = within x words, regardless of order<br>• * = truncation of word for alternate endings<br><br>Note: the *Exact search*-function was used for all the searches

#	Searches	Results
1	TS=((ATAM OR ATLS OR PTC OR PHTLS OR trauma OR "trauma care" OR "trauma team" OR "trauma team development" OR "trauma management" OR "trauma training") NEAR/0 (course$ OR "continuing education" OR "continuing professional development" OR curriculum$ OR education* OR program* OR training* OR workshop$))	109
2	TS=(trauma NEAR/3 ("continuing education" OR "education* course$" OR "education* program*" OR "training course$" OR "training program*"))	27
3	TS=(("trauma care" OR "trauma team$") AND ("continuing education" OR "education* course$" OR "education* program*" OR "training course$" OR "training program*"))	31
4	TS=(("advanced trauma life support" OR "primary trauma care" OR "trauma assessment and management" OR "trauma evaluation and management" OR "prehospital trauma life support") AND (course$ OR curriculum$ OR education* OR program* OR training* OR workshop$))	32
5	#4 OR #3 OR #2 OR #1	147
**6**	**#4 OR #3 OR #2 OR #1 and English (Languages)**	**144**

Supplementary Table 6. Cinahl Search Strategy

Interface: EBSCO<br><br>Date of Search: 11 August 2025<br><br>Number of hits: 1,728	Field labels<br><br>• MH+ = exploded Cinahl Heading<br>• MH = non exploded Cinahl Heading<br>• TI = title<br>• AB = abstract<br>• Nx = within x words, regardless of order<br>• * = truncation of word for alternate endings<br><br>Note: Expanders - Apply equivalent subjects and<br>Search modes - Find all my search terms was used for all the searches

#	Query	Results
S01	(MH "Traumatology")	890
S02	(MH "Trauma Centers")	8,181
S03	(MH "Multiple Trauma")	3,703
S04	(MH "Wounds and Injuries/TH")	5,395
S05	(MH "Advanced Trauma Life Support Care")	129
S06	S1 OR S2 OR S3 OR S4 OR S5	16,589
S07	MW "ED"	232,139
S08	(MH "Educational Measurement+")	122,981
S09	(MH "Education, Medical+")	44,175
S10	(MH "Refresher Courses")	694
S11	(MH "Educational Status")	51,261
S12	S7 OR S8 OR S9 OR S10 OR S11	394,258
S13	S6 AND S12	1,049
S14	TI (((ATAM OR ATLS OR PTC OR PHTLS OR trauma OR "trauma care" OR "trauma team" OR "trauma team development" OR "trauma management" OR "trauma training") W0 (course# OR "continuing education" OR "continuing professional development" OR curriculum# OR education* OR program* OR training* OR workshop#))) OR AB (((ATAM OR ATLS OR PTC OR PHTLS OR trauma OR "trauma care" OR "trauma team" OR "trauma team development" OR "trauma management" OR "trauma training") W0 (course# OR "continuing education" OR "continuing professional development" OR curriculum# OR education* OR program* OR training* OR workshop#)))	649
S15	TI ((trauma N3 ("continuing education" OR "education* course#" OR "education* program*" OR "training course#" OR "training program*"))) OR AB ((trauma N3 ("continuing education" OR "education* course#" OR "education* program*" OR "training course#" OR "training program*")))	152

S16	TI ((("trauma care" OR "trauma team#") AND ("continuing education" OR "education* course#" OR "education* program*" OR "training course#" OR "training program*"))) OR AB ((("trauma care" OR "trauma team#") AND ("continuing education" OR "education* course#" OR "education* program*" OR "training course#" OR "training program*")))	106
S17	TI ((("advanced trauma life support" OR "primary trauma care" OR "trauma assessment and management" OR "trauma evaluation and management" OR "prehospital trauma life support") AND (course# OR curriculum# OR education* OR program* OR training* OR workshop#))) OR AB ((("advanced trauma life support" OR "primary trauma care" OR "trauma assessment and management" OR "trauma evaluation and management" OR "prehospital trauma life support") AND (course# OR curriculum# OR education* OR program* OR training* OR workshop#)))	160
S18	S14 OR S15 OR S16 OR S17	864
S19	S13 OR S18	1,771
**S20**	**S13 OR S18 and English**	**1,728**

Supplementary Table 7. Google Scholar Search Strategy

Interface: Harzing, A.W. (2007) **Publish or Perish**, available from https://harzing.com/resources/publish-or-perish<br><br>Date of Search: 11 August 2025<br><br>Number of hits: 1,040	Search syntax:<br>• Character limit for search strings is 256 characters<br>• Phrase searching with quotation marks<br>• Does not recognize truncation symbols - Plural, genitive and other word forms will be searched automatically<br>• Field search: Title words<br>• Note: No citations or patents

#	**Searches**
#1	Title words:<br>"trauma care course" OR "trauma care courses" OR "trauma care workshop" OR "trauma course" OR "trauma courses" OR "trauma training" OR "trauma team development course" OR "trauma workshop"

Supplementary Materials Section 2:

Supplementary Table 8. The I^2 statistic, results of the Egger's regression test and the rank correlation test of the 17 studies included in the meta-analysis.

Test	Result
**Statistical tests for heterogeneity using the REML estimator in R**	
I^2 (total heterogeneity/total variability)	74.41 %
p-value of the estimate	p-val < 0.05 (p = <0.0001
χ^2 (Cochran's Q test for heterogeneity)	57.00 , Q (df =16), p-val < 0.0001
Tau^2 (estimated amount of total heterogeneity)	0.12 (SE= 0.07)
**Tests for Publication bias**	
Egger's regression test (test for Funnel Plot Asymmetry)	p = 0.1988
Rank correlation test	Kendall's tau = 0.0294, p = 0.9032

*REML- restricted maximum likelihood.

Supplementary Table 9. The results of the leave-one-out analysis of the 17 studies included in the meta-analysis.

Study	OR	CI	P-value	I^2
Vestrup et al. 1988	0.593	(0.4687 - 0.7502)	0	76.2617
Ali et al. 1993	0.6412	(0.5684 - 0.7233)	0	14.376
van Olden et al. 2004	0.6016	(0.4762 - 0.76)	0	76.2889
Wang et al. 2010	0.5821	(0.458 - 0.7398)	0	74.1752
Noordin et al. 2011	0.6004	(0.4706 - 0.766)	0	76.4089
Hashmi et al. 2013	0.6083	(0.4774 - 0.7751)	0.0001	75.9866
Hondo et al. 2013	0.598	(0.4652 - 0.7686)	0.0001	65.6049
Petroze et al. 2015	0.5771	(0.4556 - 0.7309)	0	73.0162
Bellanova et al. 2016	0.6049	(0.4796 - 0.7629)	0	76.0626
Magnone et al. 2016	0.6054	(0.4778 - 0.7672)	0	76.3348
Dennis et al. 2016	0.584	(0.4667 - 0.7307)	0	74.8399
Cioe-Pena et al. 2016	0.582	(0.4634 - 0.731)	0	74.9283
Yao et al. 2018	0.5819	(0.4579 - 0.7395)	0	74.1665
Bauman et al. 2024	0.5825	(0.4629 - 0.7331)	0	75.1027
Kamau et al. 2024	0.6093	(0.4837 - 0.7675)	0	75.6856
Nguyen et al. 2025	0.6081	(0.4762 - 0.7765)	0.0001	75.5642
Priestap et al. 2025	0.5841	(0.4648 - 0.7339)	0	75.1845

Sensitivity Analysis: Removal of Ali et al., 1993 from the study

Supplementary Table 10. The I^2 statistic, results of the Egger's regression test and the rank correlation test of the meta-analysis with Ali et al., 1993 removed.

Test	Result
**Statistical tests for heterogeneity using the REML estimator in R**	
I^2 (total heterogeneity/total variability)	14.4 %
p-value of the estimate	p-val < 0.05 (p = <0.0001)
χ^2 (Cochran's Q test for heterogeneity)	19.39 , Q (df =15), p-va1= 0.1964
Tau^2 (estimated amount of total heterogeneity)	0.01 (SE= 0.02)
**Tests for Publication bias**	
Egger's regression test (test for Funnel Plot Asymmetry)	p = 0.45
Rank correlation test	Kendall's tau = 0.0667, p = 0.7566

Supplementary Table 11. The results of the leave-one-out analysis of the meta-analysis with Ali et al., 1993 removed.

Study	OR	CI	P-value	I^2
Vestrup et al. 1988	0.641	(0.566 - 0.7258)	0	16.3048
van Olden et al. 2004	0.645	(0.5692 - 0.7309)	0	16.9684
Wang et al. 2010	0.6242	(0.56 - 0.6958)	0	6.9574
Noordin et al. 2011	0.6498	(0.5673 - 0.7442)	0	20.046
Hashmi et al. 2013	0.656	(0.5753 - 0.7479)	0	17.3834
Hondo et al. 2013	0.6633	(0.5613 - 0.7839)	0	16.6823
Petroze et al. 2015	0.6125	(0.569 - 0.6592)	0	0.0114
Bellanova et al. 2016	0.6462	(0.5704 - 0.732)	0	16.9099
Magnone et al. 2016	0.6486	(0.5709 - 0.7368)	0	17.7577
Dennis et al. 2016	0.6361	(0.5661 - 0.7148)	0	13.1211
Cioe-Pena et al. 2016	0.6346	(0.5649 - 0.713)	0	12.6733
Yao et al. 2018	0.6239	(0.5602 - 0.6949	0	6.7168
Bauman et al. 2024	0.6346	(0.5644 - 0.7136)	0	12.8695
Kamau et al. 2024	0.6484	(0.5723 - 0.7345)	0	16.8911
Nguyen et al. 2025	0.659	(0.5775 - 0.7519)	0	16.2779
Priestap et al. 2025	0.6359	(0.5652 - 0.7153)	0	13.3549

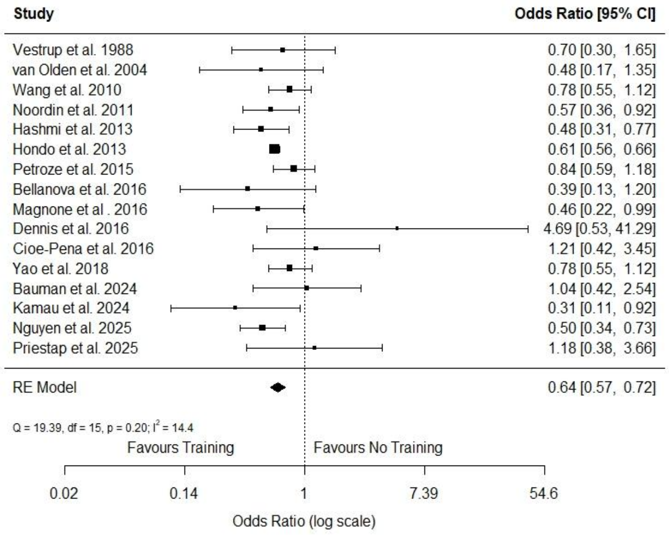

Supplementary Figure 1. Forest plot of the meta-analysis with Ali et al., 1993 removed.

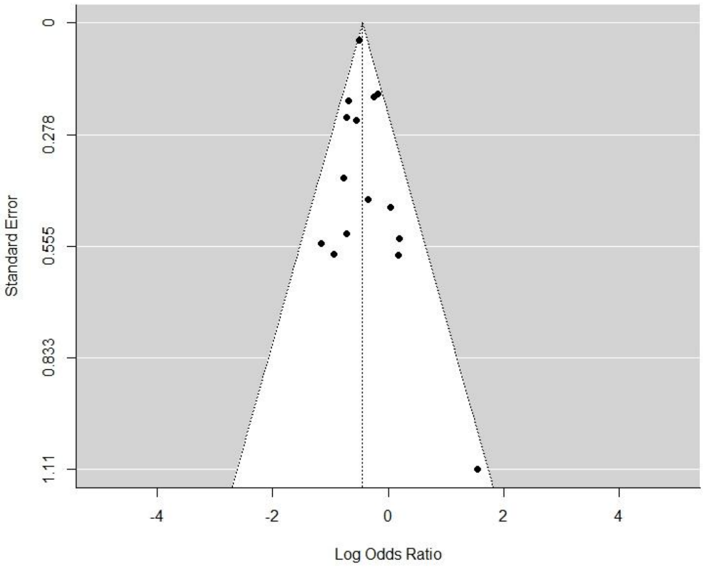

Supplementary Figure 2. Funnel plot of the meta-analysis with Ali et al., 1993 removed.

Sensitivity Meta-Analysis: Advanced Trauma Life Support (ATLS)

Supplementary Table 12. The I^2 statistic, results of the Egger's regression test and the rank correlation test of the studies on ATLS.

Test	Result
**Statistical tests for heterogeneity using the REML estimator in R**	
I^2 (total heterogeneity/total variability)	69.1 %
p-value of the estimate	p-val < 0.05 (p = < 0.0001)
χ^2 (Cochran's Q test for heterogeneity)	40.03 , Q (df =9), p-val < 0.0001
Tau^2 (estimated amount of total heterogeneity)	0.15 (SE= 0.11)
**Tests for Publication bias**	
Egger's regression test (test for Funnel Plot Asymmetry)	p = 0.72
Rank correlation test	Kendall's tau = -0.2889, p = 0.2912

Supplementary Table 13. The results of the leave-one-out analysis of the studies on ATLS.

Study	OR	CI	P-value	I^2
Vestrup et al. 1988	0.495	(0.3556 - 0.6891)	0	72.0303
Ali et al. 1993	0.6202	(0.4985 - 0.7715)	0	22.2708
van Olden et al. 2004	0.5095	(0.3667 - 0.7079)	0.0001	72.421
Wang et al. 2010	0.4743	(0.3406 - 0.6604)	0	65.5023
Noordin et al. 2011	0.4995	(0.353 - 0.707)	0.0001	71.3875
Hashmi et al. 2013	0.511	(0.3606 - 0.7241)	0.0002	71.3417
Petroze et al. 2015	0.4682	(0.3393 - 0.6461)	0	62.9436
Bellanova et al. 2016	0.5158	(0.3725 - 0.7142)	0.0001	72.1304
Magnone et al. 2016	0.5122	(0.3659 - 0.7171)	0.0001	72.4294
Kamau et al. 2024	0.523	(0.3785 - 0.7228)	0.0001	71.6041

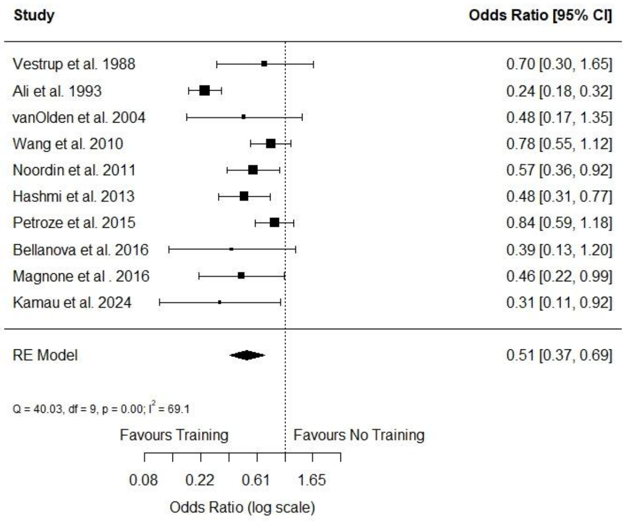

Supplementary Figure 3. Forest plot of the studies that used ATLS as an intervention.

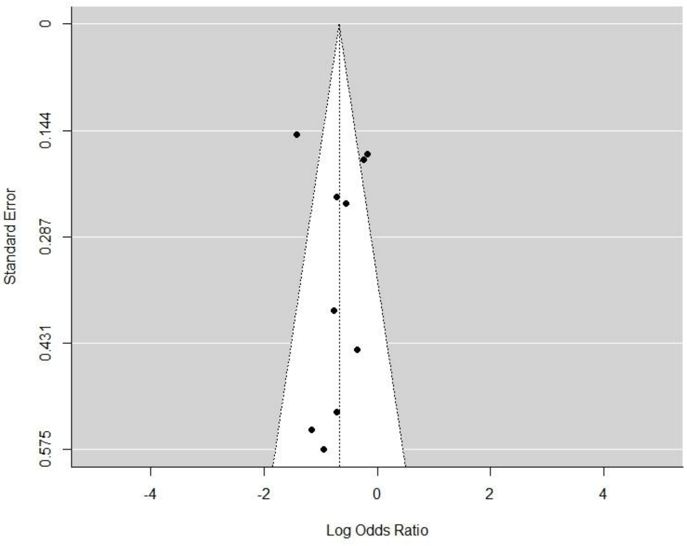

Supplementary Figure 4. Funnel plot of the studies that used ATLS as an intervention.

Sensitivity Meta-Analysis: Rural Trauma Team Development Course (RTTDC)

Supplementary Table 14. The I^2 statistic, results of the Egger's regression test and the rank correlation test of the studies on RTTDC.

Test	Result
**Statistical tests for heterogeneity using the REML estimator in R**	
I^2 (total heterogeneity/total variability)	0.0 %
p-value of the estimate	p-val >0.05 (p = 0.5123)
χ^2 (Cochran's Q test for heterogeneity)	1.5962 , Q (df =2), p-val = 0.4502
Tau^2 (estimated amount of total heterogeneity)	0.00 (SE= 0.37)
**Tests for Publication bias**	
Egger's regression test (test for Funnel Plot Asymmetry)	p = 0.21
Rank correlation test	Kendall's tau = 1.0000, p = 0.3333

Supplementary Table 15. The results of the leave-one-out analysis of the studies on RTTDC.

Study	OR	CI	P-value	I^2
Dennis et al. 2016	1.0895	(0.5398 - 2.1993)	0.8109	0
Bauman et al. 2024	1.6982	(0.5163 - 5.5855)	0.3833	17.6351
Priestap et al. 2025	1.5701	(0.4187 - 5.8874)	0.5035	36.7526

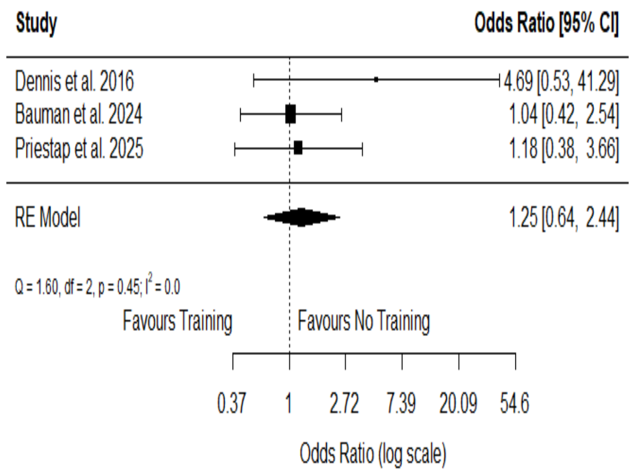

Supplementary Figure 5. Forest plot of the studies that used RTTDC as an intervention.

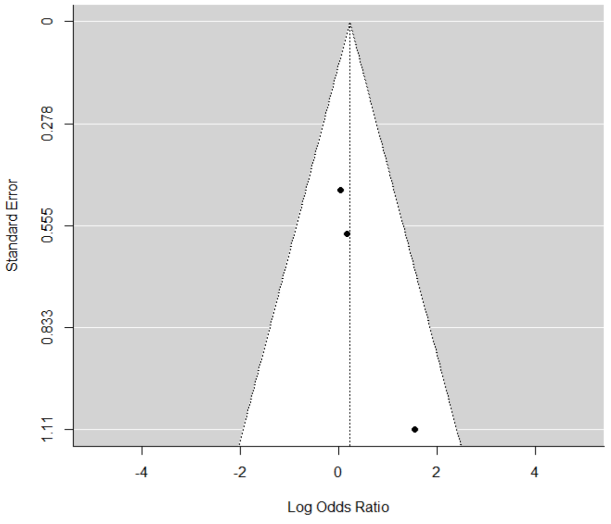

Supplementary Figure 5. Funnel plot of the studies that used RTTDC as an intervention.

Sensitivity Meta-Analysis: Studies that measured mortality at less than 1 week

Supplementary Table 16. The I^2 statistic, results of the Egger's regression test and the rank correlation test of the studies that measured mortality at < 1 week.

**Test**	**Result**
**Statistical tests for heterogeneity using the REML estimator in R**	
I^2 (total heterogeneity/total variability)	0.0 %
p-value of the estimate	p-val <0.05 (p = <0.0001)
χ^2 (Cochran's Q test for heterogeneity)	0.38 , Q (df =2), p-val = 0.8255
Tau^2 (estimated amount of total heterogeneity)	0.00 (SE= 0.17)
**Tests for Publication bias**	
Egger's regression test (test for Funnel Plot Asymmetry)	p = 0.60
Rank correlation test	Kendall's tau = 0.3333, p = 1.0000

Supplementary Table 17. The results of the leave-one-out analysis of the studies that measured mortality at < 1 week.

**Study**	**Estimate**	**CI**	**P-value**	I^2
van Olden et al. 2004	0.3489	(0.2337 - 0.5211)	0	0
Bellanova et al. 2016	0.3615	(0.2433 - 0.5371)	0	0
Nguyen et al. 2025	0.4389	(0.2055 - 0.9374)	0.0334	0

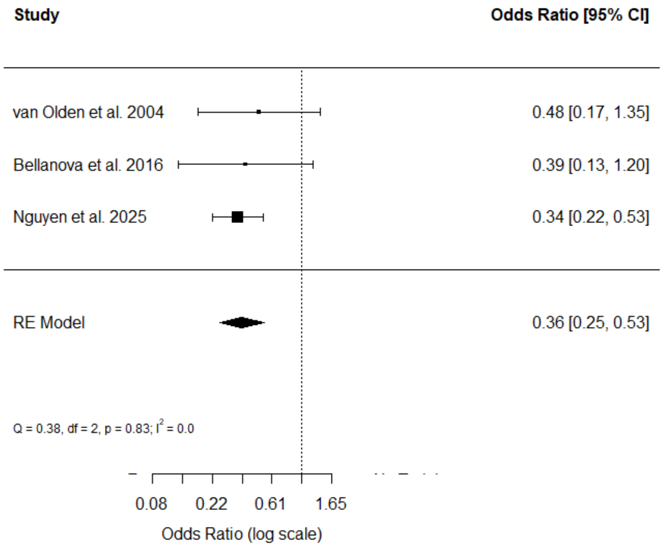

Supplementary Figure 7. Forest plot of the studies that of the studies that measured mortality at < 1 week.

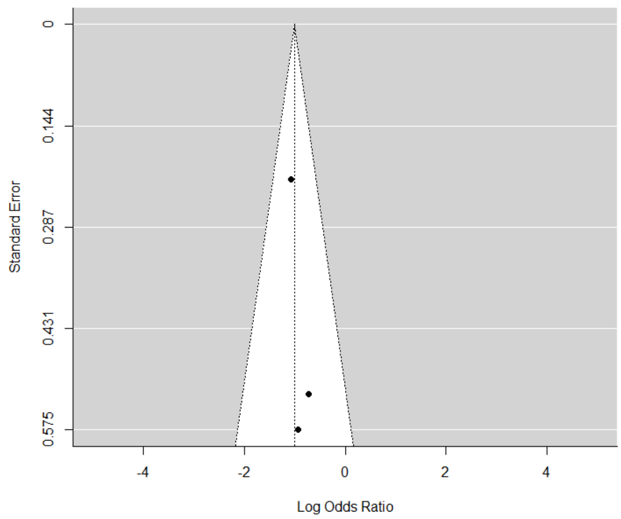

Supplementary Figure 8. Funnel plot of the studies that of the studies that measured mortality at < 1 week.

Sensitivity Meta-Analysis: Studies that measured in-hospital mortality

Supplementary Table 18. The I^2 statistic, results of the Egger's regression test and the rank correlation test of the studies that measured in-hospital mortality.

Test	Result
**Statistical tests for heterogeneity using the REML estimator in R**	
I^2 (total heterogeneity/total variability)	82.9 %
p-value of the estimate	p-val <0.05 (p = 0.0071)
χ^2 (Cochran's Q test for heterogeneity)	49.9 , Q (df =10), p-val < 0.001
Tau^2 (estimated amount of total heterogeneity)	0.18 (SE= 0.12)
**Tests for Publication bias**	
Egger's regression test (test for Funnel Plot Asymmetry)	p = 0.02
Rank correlation test	Kendall's tau = 0.2364, p = 0.3587

Supplementary Table 19. The results of the leave-one-out analysis of the of the studies that measured in-hospital mortality.

Study	Estimate	CI	P-value	I^2
Vestrup et al. 1988	0.6505	(0.4627 - 0.9145)	0.0133	85.3567
Ali et al. 1993	0.6669	(0.5734 - 0.7757)	0	19.9165
Wang et al. 2010	0.6391	(0.4492 - 0.9094)	0.0128	83.5129
Noordin et al. 2011	0.6688	(0.468 - 0.9557)	0.0272	85.2982
Hashmi et al. 2013	0.681	(0.4784 - 0.9695)	0.033	84.8054
Hondo et al. 2013	0.6683	(0.4624 - 0.9659)	0.032	75.9634
Dennis et al. 2016	0.6222	(0.4571 - 0.847)	0.0026	83.1572
Cioe-Pena et al. 2016	0.6239	(0.4527 - 0.86)	0.004	83.6885
Yao et al. 2018	0.6388	(0.4491 - 0.9088)	0.0127	83.5136
Bauman et al. 2024	0.6273	(0.4524 - 0.8699)	0.0052	84.0444
Priestap et al. 2025	0.6276	(0.4549 - 0.866)	0.0046	83.9529

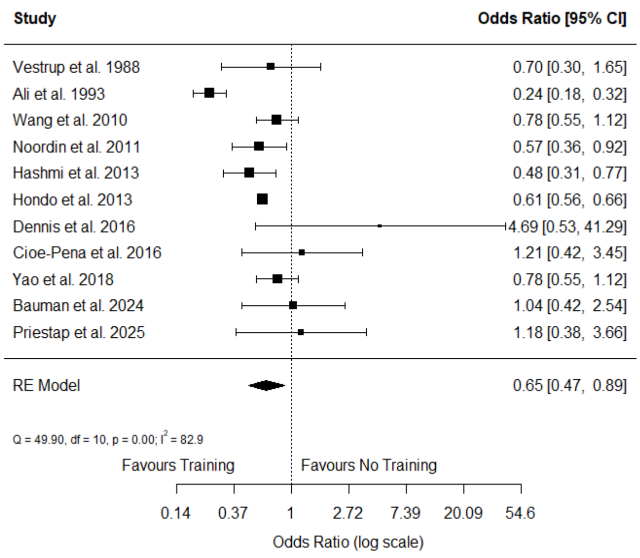

Supplementary Figure 9. Forest plot of the studies that of the studies that measured in-hospital mortality.

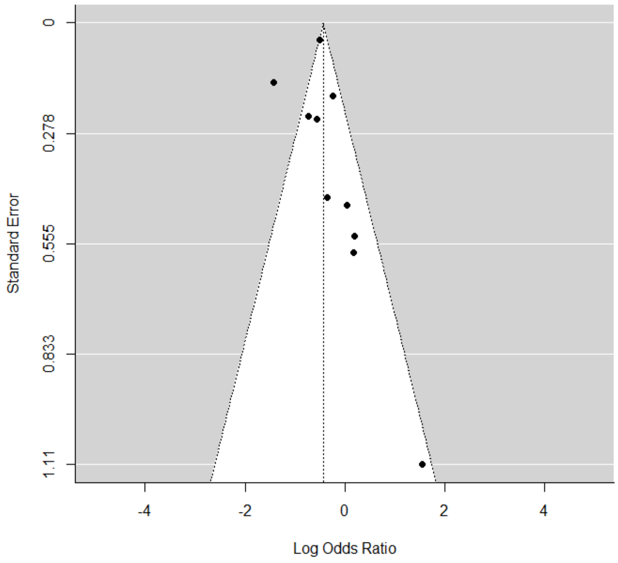

Supplementary Figure 10. Funnel plot of the studies that of the studies that measured in-hospital mortality.

Sensitivity Meta-Analysis: Studies that measured 30-day mortality

Supplementary Table 20. The I^2 statistic, results of the Egger's regression test and the rank correlation test of the studies that measured mortality at 30-days.

Test	Result
**Statistical tests for heterogeneity using the REML estimator in R**	
I^2 (total heterogeneity/total variability)	65.0 %
p-value of the estimate	p-val <0.05 (p = 0.0284)
χ^2 (Cochran's Q test for heterogeneity)	5.69 , Q (df = 2), p-val = 0.06
Tau^2 (estimated amount of total heterogeneity)	0.11 (SE= 0.18)
**Tests for Publication bias**	
Egger's regression test (test for Funnel Plot Asymmetry)	p = 0.25
Rank correlation test	Kendall's tau = -0.3333, p = 1.0000

Supplementary Table 21. The results of the leave-one-out analysis of the studies that measured mortality at 30-days.

Study	Estimate	CI	P-value	I^2
Petroze et al. 2015	0.4738	(0.3319 - 0.6765)	0	0
Kamau et al. 2024	0.6496	(0.3913 - 1.0782)	0.0952	74.646
Nguyen et al. 2025	0.5888	(0.235 - 1.4749)	0.2582	65.3799

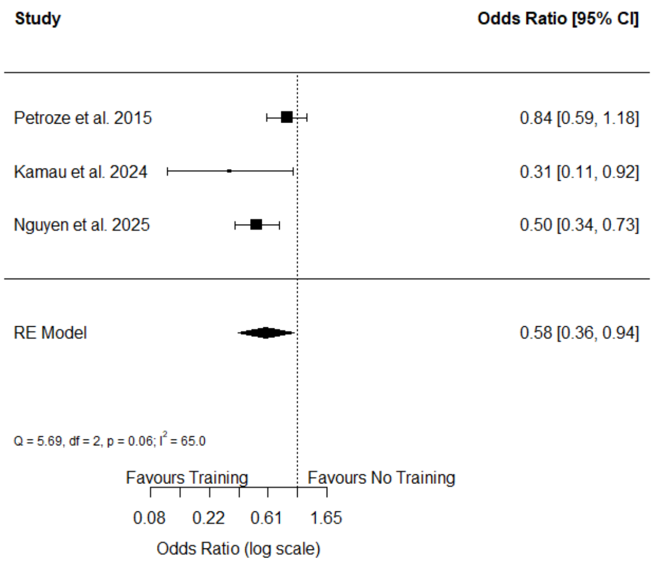

Supplementary Figure 11. Forest plot of the studies that of the studies that measured mortality at 30-days.

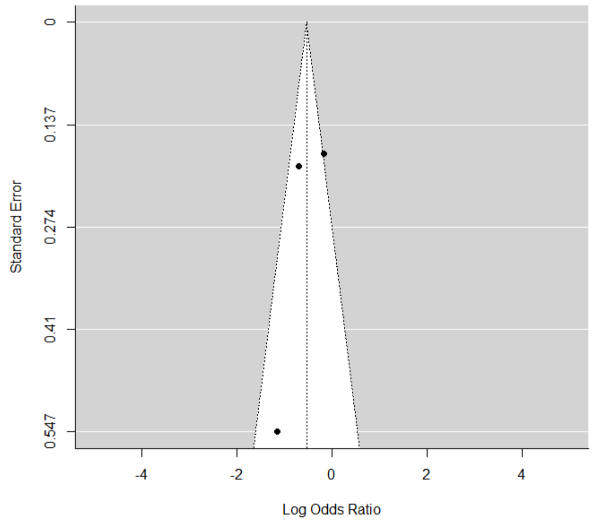

Supplementary Figure 12. Funnel plot of the studies that of the studies that measured mortality at 30-days.

Sensitivity Meta-Analysis: Studies that are set in high-income countries

Supplementary Table 22. The I^2 statistic, results of the Egger's regression test and the rank correlation test of the studies set in high-income countries.

**Test**	**Result**
**Statistical tests for heterogeneity using the REML estimator in R**	
I^2 (total heterogeneity/total variability)	0.0 %
p-value of the estimate	p-val <0.05 (p = <0.0001)
χ^2 (Cochran's Q test for heterogeneity)	7.46 , Q (df = 7), p-val = 0.38
Tau^2 (estimated amount of total heterogeneity)	0.00 (SE= 0.04)
**Tests for Publication bias**	
Egger's regression test (test for Funnel Plot Asymmetry)	p = 0.31
Rank correlation test	Kendall's tau = 0.3571, p = 0.2751

Supplementary Table 23. The results of the leave-one-out analysis of the studies set in high-income countries.

**Study**	**Estimate**	**CI**	**P-value**	I^2
Vestrup et al. 1988	0.6079	(0.5603 - 0.6595)	0	0.0022
van Olden et al. 2004	0.6095	(0.5618 - 0.6613)	0	0.0051
Hondo et al. 2013	0.6773	(0.465 - 0.9866)	0.0423	0.0003
Bellanova et al. 2016	0.6101	(0.5623 - 0.6618)	0	0.0037
Magnone et al. 2016	0.6106	(0.5627 - 0.6625)	0	0.0001
Dennis et al. 2016	0.6069	(0.5596 - 0.6582)	0	0
Bauman et al. 2024	0.606	(0.5585 - 0.6575)	0	0.0026
Priestap et al. 2025	0.6066	(0.5591 - 0.658)	0	0.0033

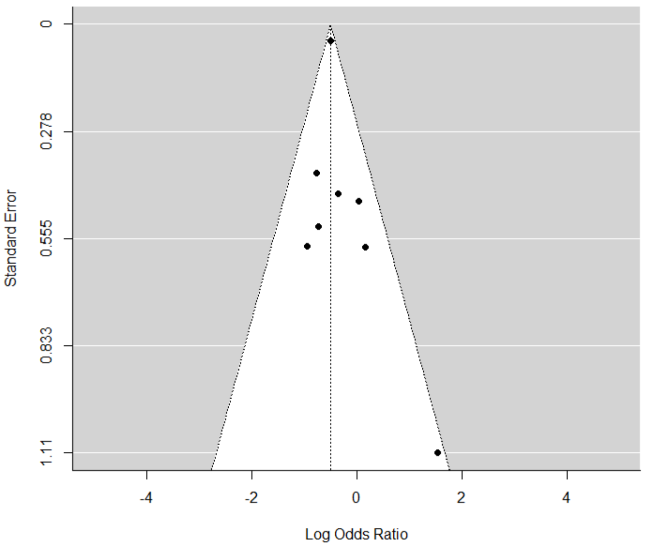

Supplementary Figure 13. Forest plot of the studies that of the studies set in high-income countries.

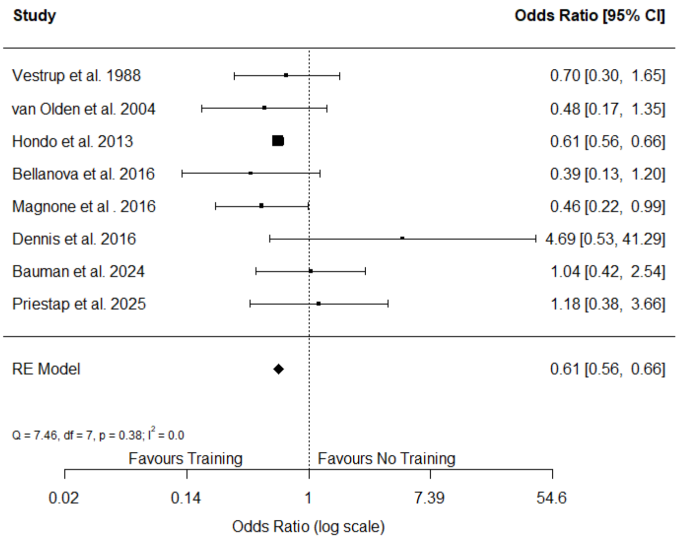

Supplementary Figure 14. Funnel plot of the studies that of the studies set in high-income countries.

Sensitivity Meta-Analysis: Studies that are set in upper-middle-income countries

Supplementary Table 24. The I^2 statistic, results of the Egger's regression test and the rank correlation test of the studies set in upper-middle-income countries.

Test	Result
**Statistical tests for heterogeneity using the REML estimator in R**	
I^2 (total heterogeneity/total variability)	90.9 %
p-value of the estimate	p-val >0.05 (p = 0.1536)
χ^2 (Cochran's Q test for heterogeneity)	38.21 , Q (df = 3), p-val < 0.001
Tau^2 (estimated amount of total heterogeneity)	0.41 (SE= 0.400)
**Tests for Publication bias**	
Egger's regression test (test for Funnel Plot Asymmetry)	p = 0.27
Rank correlation test	Kendall's tau = 0.3333, p = 0.7500

Supplementary Table 25. The results of the leave-one-out analysis of the studies set in upper-middle-income countries.

Study	OR	CI	P-value	I^2
Ali et al. 1993	0.8018	(0.6261 - 1.0268)	0.08	0
Wang et al. 2010	0.5668	(0.2198 - 1.4615)	0.2401	92.4903
Cioe-Pena et al. 2016	0.5258	(0.2427 - 1.1393)	0.1032	93.6942
Yao et al. 2018	0.5662	(0.2198 - 1.4589)	0.2389	92.5033

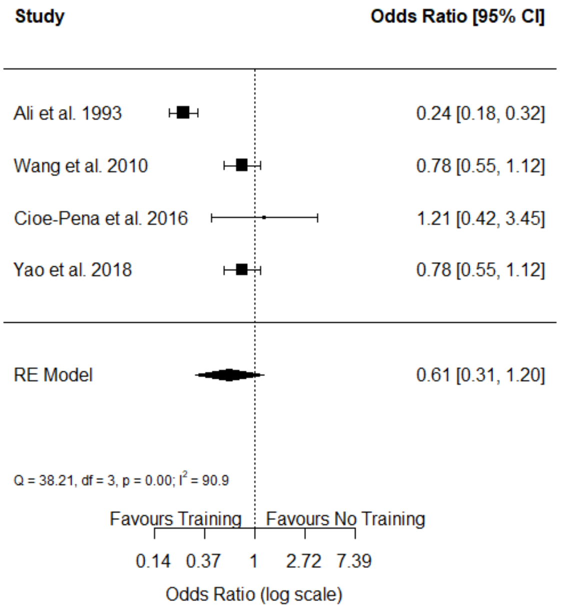

Supplementary Figure 15. Forest plot of the studies set in upper-middle-income countries.

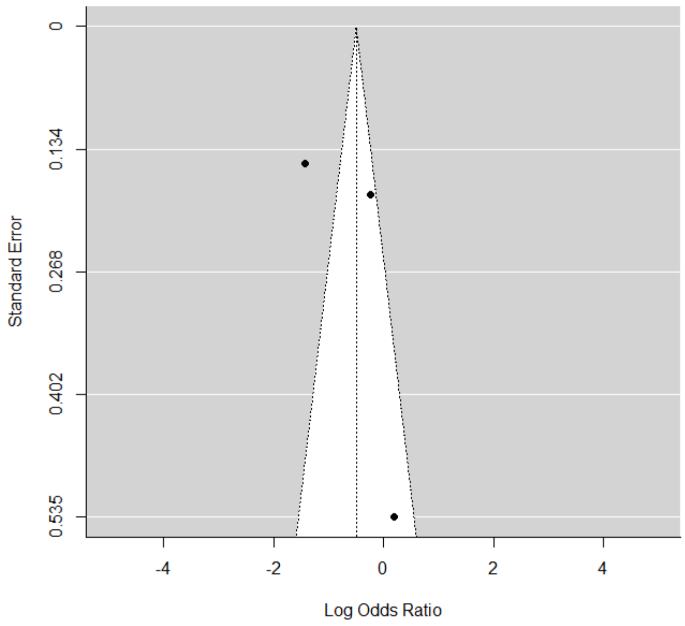

Supplementary Figure 16. Funnel plot of the studies that of the studies set in upper-middle-income countries.

Sensitivity Meta-Analysis: Studies that are set in lower-middle-income countries

Supplementary Table 26. The I^2 statistic, results of the Egger's regression test and the rank correlation test of the studies set in lower-middle-income countries.

Test	Result
**Statistical tests for heterogeneity using the REML estimator in R**	
I^2 (total heterogeneity/total variability)	0.0 %
p-value of the estimate	p-val <0.05 (p = 0.0001)
χ^2 (Cochran's Q test for heterogeneity)	1.03 , Q (df = 3), p-val = 0.79
Tau^2 (estimated amount of total heterogeneity)	0.00 (SE= 0.05)
**Tests for Publication bias**	
Egger's regression test (test for Funnel Plot Asymmetry)	p = 0.45
Rank correlation test	Kendall's tau = -0.3333, p = 0.7500

Supplementary Table 27. The results of the leave-one-out analysis of the studies set in lower-middle-income countries.

Study	Estimate	CI	P-value	I^2
Noordin et al. 2011	0.4778	(0.3607 - 0.6328)	0	0
Hashmi et al. 2013	0.5067	(0.3812 - 0.6737)	0	0
Kamau et al. 2024	0.513	(0.4002 - 0.6575)	0	0
Nguyen et al. 2025	0.5017	(0.3662 - 0.6874)	0	0

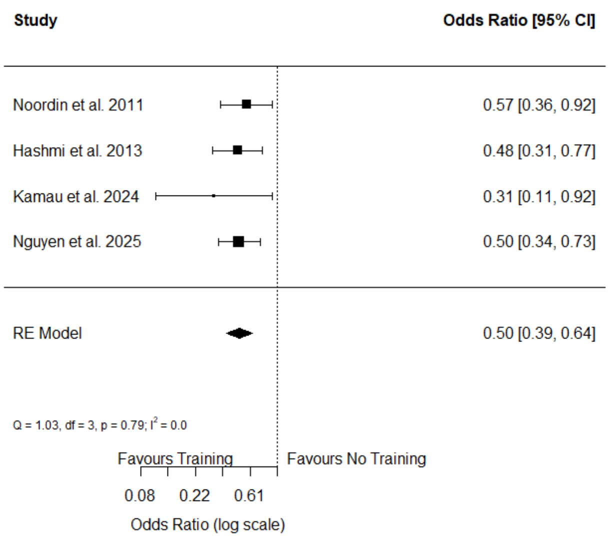

Supplementary Figure 17. Forest plot of the studies set in lower-middle-income countries.

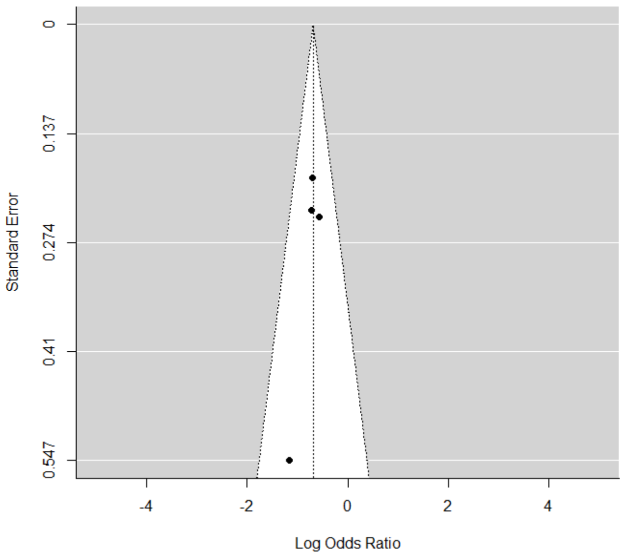

Supplementary Figure 18. Funnel plot of the studies set in lower-middle-income countries.

Supplementary Table 28. Results of the sensitivity analysis test on training programmes, outcome definitions, and country income levels.

Sensitivity analysis	Number of articles	Pooled Odds Ratio (95% CI)
**Training programme**		
ATLS (Add citations)	10	0.51 (0.37 - 0.69)
RTTDC (Add citations)	3	01.25 (0.64 - 2.44)
**Outcome definition**		
Mortality < 1 week (Add citations)	3	0.36 (0.25 - 0.53)
In-hospital mortality (Add citations)	11	0.65 (0.47 - 0.89)
30-day mortality (Add citations)	3	0.58 (0.36 - 0.94)
**Country income level***		
HICs (Add citations)	8	0.61 (0.56 -0.66)
UMICs (Add citations)	5	0.61 (0.31 -1.20)
LMICs (Add citations)	4	0.50 (0.39 -0.64)

*Country income level refers to the income level of the country where the studies were conducted.
Abbreviations: ATLS- Advanced Trauma Life Support Training, HICs- High-income countries, LMICs- Lower-middle income countries, RTTDC- Rural Trauma Team Development Course, UMICs- Upper-middle income countries.
